# COVID-19 and Women's Health: A Low- and Middle-Income Country Perspective

**DOI:** 10.3389/fgwh.2020.572158

**Published:** 2020-10-23

**Authors:** Shahirose Sadrudin Premji, Kiran Shaikh, Sharifa Lalani, Ilona S. Yim, Sarah Moore, Naureen Akber Ali, Saher Aijaz, Nicole Letourneau

**Affiliations:** ^1^Faculty of Health, School of Nursing, York University, Toronto, ON, Canada; ^2^School of Nursing and Midwifery, Aga Khan University, Karachi, Pakistan; ^3^Department of Psychological Science, University of California, Irvine, Irvine, CA, United States; ^4^Department of Medical Genetics, Faculty of Medicine, University of British Columbia, Vancouver, BC, Canada; ^5^Faculty of Nursing, University of Calgary, Calgary, AB, Canada

**Keywords:** COVID-19, pregnancy, anxiety, depression, stress, mental health, preterm birth, low- and middle-income countries

## Abstract

Corona Virus Disease (COVID-19), a contagious disease, is a global pandemic affecting the lives and health of individuals across borders, genders and races. Much of what is known about the effects of natural disasters and disease outbreaks on women's health in particular, is based on studies conducted in high-income countries. The evolving evidence suggests that COVID-19 has a profound negative impact on the perinatal mental health of women. It is also clear that global pandemics such as COVID-19 disproportionately affect the less affluent, including individuals living in low- and middle-income countries. The purpose of this review is to summarize and critically discuss extant knowledge on COVID-19 as it relates to the perinatal health of women in low and middle-income countries, using Pakistan as a case example. We specifically highlight the effects on perinatal mental health, preterm birth, and timing of the COVID-19 exposure. Our review suggests that it is essential to consider the effects of COVID-19 within this cultural context and that findings from high-income countries do not necessarily translate to the situation in low and middle-income countries.

## Background

Corona Virus Disease (COVID-19), a new ribonucleic acid virus which presents with symptoms of fever tiredness, and dry cough (1), was declared a global pandemic by the World Health Organization (1, 2). Empirical evidence suggests that natural disasters and disease outbreaks elicit a profound negative impact on the perinatal health outcomes in part due to excessive exposure to distressing environmental situations (3–5). With a global pre-pandemic prevalence of 20 percent (6, 7), perinatal mental distress (i.e., anxiety, depression, and stress during pregnancy and postpartum) already imposes a significant threat to women's health (physical and emotional) and perinatal outcomes (8–10). Furthermore, the likelihood of adverse cognitive, behavioral, and emotional outcomes also increases in children born to these mothers (11). Much of this empirical evidence on natural disasters and disease outbreaks is based in high-income countries limiting our understanding of the impact of disease outbreaks on perinatal health outcomes of women residing in low- and middle-income (LMI) countries (3). Moreover, the physiological and cellular-level impacts of COVID-19 on pregnant women have not been studied (12–14). In this concise summary we address the existing knowledge, vastly informed by studies on (a) COVID-19 primarily from China, (b) infectious diseases (disaster and pandemic influenza) predominantly based in high-income countries, and (c) perinatal mental health of women of which only 8–15% of the studies are from LMI countries (compared to 90% from high-income countries) (15). We situate this review within the context of an LMI country like Pakistan.

## COVID-19 Within the Context of Pakistan

COVID-19 reached Pakistan in February 2020 and a nationwide lockdown was initiated on March 23, 2020 (16). Data from Worldometer info indicates that Pakistan ranks 17 among 213 countries and territories around the word. The number of cases in Pakistan is increasing exponentially with total cases of 103,671 (as of June 7, 2020) among a population of roughly 220 million. The province of Punjab has the highest number of total case. Karachi, which is in the Sindh province has the second highest number of COVID-19 cases in Pakistan (16). Although Sindh is the third largest province, 48 million people with diverse ethnic and religious backgrounds reside there making it the second largest populated province in Pakistan (17). The incidence of COVID-19 in Pakistani perinatal women will vary depending on the number of people per unit area and demographics, and availability of testing and reporting mechanisms (18), which at present is limited in Pakistan.

## COVID-19 and Mental Health of Pakistani Pregnant Women

The pandemic has increased women's anxiety and stress levels, and depressive symptoms (19, 20) and those who are pregnant reported increased worry and fears (i.e., perinatal mental distress) regarding their own, their baby's and their family members' health (21). Albeit limited (4), evidence underlying the impact of pandemic situations on the mental health of pregnant women, suggests an additional burden (22) given the uncertainty regarding disease susceptibility, vertical transmission to unborn baby/newborn, and management of the infected pregnant women (23).

Haider (22) in the article published in The Express Tribune narrated a story of a 27-years old women, 12-weeks pregnant, from the North-East of Karachi who described her level of anxiety as “going through the roof” further adding “Since then, I have not only been fearing for my own life but also for my unborn baby. This feeling of uncertainty is killing me.” In a survey undertaken by *The Express Tribune*, 62% of 110 pregnant women across Pakistan, aged 25–30 years, were apprehensive about antenatal visits at hospital or clinics (22). Their primary worry was contracting COVID-19 while in the hospital with secondary fears related to exposure to COVID-19 patients in the hospital (22). Fear and worries were situated in pregnancy as evident from statements such as “How [will I] take care of the baby? Will the hospital be taking mine and the baby's hygiene seriously as they already have so many COVID-19 cases to deal with?” (22) Pregnancy during the pandemic elicited mixed feelings “New additions to the family are a time of celebration and while it still is going to be a celebration, there is still a worry to keep the baby safe—more than ever now” (22).

Accurate health information and engaging in practices to prevent/stop COVID-19 (e.g., washing hands, wearing a mask) lowered stress, anxiety, and depression among women (19). COVID-19 will likely have a disproportionate toll on Pakistani pregnant women's perinatal mental health due to low literacy levels (24), inequities in access to basic needs (e.g., water) and protective equipment given socio-economic determinants of health (25). COVID-19 has altered how women give birth with health care systems instituting varied approaches to care provision during labor and delivery (e.g., no or only one support person). The COVID-19 pandemic limits women's ability to anticipate and form realistic expectations of labor and delivery thereby impacting their readiness mentally and physically, increasing the likelihood of viewing the experience as traumatic (even when their pain is well-managed, or they deliver a healthy baby.

A study examining the psychosocial effect of the Severe Acute Respiratory Syndrome (SARS) outbreak noted worsened mental health and posttraumatic stress disorder among women more so than men (26). Given the vulnerability of Pakistani women, particularly pregnant and postpartum women to post-disaster mental health issues (3), it is imperative to understand women's lived experience to identify strategies to alleviate the negative and unintended consequences of preventive strategies such as social distancing, and self-isolation (27). COVID-19- specific public health strategies such as social distancing and staying home will have social and economic repercussions raising concerns regarding the mental health and social well-being of the general population (28). Across all LMI countries, women and children are the most vulnerable to inequities in socio-economic determinants of health (25) which will be magnified in a pandemic situation. An alarming increase in domestic violence has been reported globally (29). An online mental health counseling service provider in Pakistan indicated an upsurge in domestic violence cases, and psychological health issues amidst lockdown, social isolation and economic crisis (30). COVID-19 pandemic has placed a strain on healthcare systems globally (28), which are primarily focused on the management of outbreak during this time. As a result, antenatal and postnatal care, and mental health services may have been hampered during this time.

## COVID-19 and Preterm Birth In Pakistan

In Pakistan 16% of infants are born preterm every year, representing the world's highest rate of preterm birth, a sharp contrast from 8% in a high-income country like Canada (31, 32). The social, cultural, and environmental context of LMI countries like Pakistan produces more extreme and prolonged exposure to stressors (i.e., chronic stress) (33, 34), inducing greater perinatal mental distress (35) and increased risk of preterm birth. The effect of perinatal mental distress on preterm birth however varies by location (high vs. low-income countries) (36), socioeconomic status (36), types of perinatal mental distress, and periods of gestation (37, 38). Pandemic stress or possible COVID-19 infection may exacerbate rates of preterm birth or complicate care as pregnant women's susceptibility to coronavirus, transmission to newborn, and clinical presentation and management when infected remain unknown (23, 39). Risk of preterm birth may be higher amongst Pakistani pregnant women exposed to COVID-19 related stress early in pregnancy.

Both animal and human studies demonstrate that psychological and biological responses to psychosocial distress vary across pregnancy, with the magnitude of responses being more pronounced early in pregnancy (37, 40, 41). Alterations in the stress response may be adaptive to protect the fetus and mother from adverse health consequences (37). The physiological and cellular-level impacts of COVID-19 on pregnant women are unknown; however, previous laboratory studies indicated histopathological and behavioral impacts of prenatal influenza infection in the offspring of mice (12, 13). Consequently, pregnant women who are exposed to the stress of COVID-19 later in pregnancy may have less psychosocial distress, dampened physiological responses, and vulnerability to preterm birth (40, 42–44). The physiological and cellular-level impacts of COVID-19 on pregnant women are, however, unknown (12, 13).

## COVID-19 and Epigenetics

Deoxyribonucleic methylation (DNAm) is an epigenetic marker that fluctuates with development and experience, yet, maintains patterns that define the identity of cells and tissues (45). Thus, DNAm lends insights to biological function (e.g., current immune response), as well as how it has adapted to experiences of stress over time, and these properties have made it particularly informative for understanding how stressors “get under the skin” in the way it influences brain development (e.g., regulation of emotion) and behavior (46). For the biological effects of prenatal experiences, especially, DNAm is particularly relevant, as massive epigenetic waves of regulation occur in the prenatal period (46, 47). Exposures, such as COVID-19 related stress, can disrupt critical developmental events *in utero* and exert lasting biological consequences *via* DNAm and other epigenetic alterations. In addition to capturing exposure to prenatal distress (48), DNAm patterns also reflect very early postnatal experiences, and DNAm in genes associated with the stress response (i.e., genes involved in the hypothalamic-pituitary-adrenal axis or HPA) prospectively predict the onset of depression (49, 50). The biological significance of COVID-19-related stress in mothers and links to preterm birth remain unknown.

## Discussion

LMI countries, like Pakistan, are characterized by large and densely populated urban regions, fragile health care infrastructure or health systems, resource capacity, water and electricity supplies), economic and social conditions (e.g., multigenerational households in small spaces), and inconsistent availability of COVID-19 testing. These characteristics create a milieu for a very high incidence and prevalence of COVID-19 infection, likely more than the current estimates indicate given data quality, affecting millions of pregnant and childbearing women and their children. Disruptions in routine antenatal care will have a disproportionate toll on maternal and newborn mortality and morbidity (51).

Like past infectious outbreaks, the likely mental health consequences during pregnancy are high, and especially so in already stressed populations as found in LMI countries. The COVID-19 pandemic has led to changes in labor and delivery practices and policies which limits the degree of social support available to women during and following childbirth. Lack of social support can heighten anxiety, delay the progression of labor, and affect the overall psychological well-being of women (52).

Past evidence on MERS-CoV and SARS-CoV also suggests that prenatal mental health of women needs to be prioritized as there is significant knowledge gap to inform practice and policies (4). COVID-19 likely increases risk for preterm birth, linked to stress (23, 39), however impacts may be less in women with advanced gestational age. To limit unintended adverse consequences of COVID-19, and pandemics in general, mental health of pregnant women should be a public health concern with care providers, researcher, and policy decisions makers asking “how are we safeguarding the short- and longer-term mental health of pregnant women and their partners in the age of coronavirus” (4).

Finally, understanding how infectious outbreaks like COVID-19 may impact the fetus' epigenome are unknown and may lead to understanding risk for preterm birth and later child health consequences. The COVID-19 pandemic has arisen at a time where the collection of biological samples for genomics analysis are commonplace due to reduced costs of genome-wide technologies, offering an unprecedented opportunity to explore how the widespread effects of pandemic stress become biologically embedded.

## Conclusion

This brief review provides some important insights into the differential ways in which COVID-19 influences women in high income vs. low-and-middle-income countries. First, the higher population density in areas such as Karachi makes exposure to COVID-19 more likely than it is in most high-income countries. Second, educational (low literacy) and economical (access to basic needs, protective equipment) constraints affect how women give birth and how much anxiety and stress they may feel about safely giving birth under the current conditions. Third, for individuals living in LMI countries, the acute stress of a pandemic occurs within an already much more stressful living situation characterized by significant poverty, thus worsening the effects of stress on perinatal health. This exacerbated stress can have detrimental downstream consequences, including preterm birth and later child health consequences. In this regard, epigenetics offers a window of opportunity to understand biological significance of COVID-19-related stress.

## Author Contributions

SP provided the concept, drafted the manuscript with contributions from KS, SL, IY, SM, and NL. SP and SA revised the manuscript. All authors approved the final version for publication.

## Conflict of Interest

The authors declare that the work was conducted in the absence of any commercial or financial relationships that could be construed as a potential conflict of interest.

## Abbreviations

COVID-19: coronavirus disease 2019
LMI: low- and middle-income
DNAm: Deoxyribonucleic methylation.
